# Experience of a Final Year Medical Student: Pre- and Post-COVID-19 Era

**DOI:** 10.31729/jnma.6353

**Published:** 2021-07-31

**Authors:** Prinsa Shrestha

**Affiliations:** 1 Kathmandu Medical College and Teaching Hospital, Sinamangal, Kathmandu, Nepal

**Keywords:** *clinical competence*, *COVID-19*, *learning*, *medical education*, *medical students*

## Abstract

Medical education provides both knowledge and clinical skills to students. Clinical skills program including bedside teaching is considered an irreplaceable part of the undergraduate medical curriculum. COVID-19 pandemic has halted the delivery of effective clinical skills to medical students which has especially affected the final year students. So, we need to find an alternate approach to teach clinical skills to medical students in this era of COVID-19. This public health crisis has also demonstrated the significance of resilience and adaptability in the medical education system and the need to inculcate these values in our generation of medical students. This will help the students to complete their transition from a 'student' to a 'doctor'. This article highlights the experience of a final year medical student in the pre- and post-COVID-19 period, problems faced by final year medical students during this crisis, and effective ways to cope up with them.

## INTRODUCTION

Medical education involves various facets and provides both knowledge and clinical skills to students.^1^ The core of medical teaching consists of knowledge, attitude, and skills which can be acquired through a mixture of various means like bedside teaching, lectures, simulation, etc.^2^ Clinical rotation provides the experience of interacting with patients; clinical reasoning; witnessing procedures; professionalism.^3^ Thus, it is an imperative aspect of the undergraduate medical curriculum. COVID-19 pandemic has halted the delivery of effective clinical skills which has especially affected the final year students; as the majority of this year is clinical with very few lectures scattered throughout the year.^2^

## PRE-COVID ERA MY LEARNING EXPERIENCE

At the beginning of my final year, I had a plan for the entire year ahead intending to make the best of the clinical postings. I had planned to review all the topics taught to us in theory as well as practical sessions on the same day. During this semester at Kathmandu Medical College, we had theory classes in the lecture hall and clinical posting in bedside and tutorial rooms. The clinical posting includes interacting with the patients, building rapport, taking history, conducting a physical examination, and deriving provisional diagnosis.^3^ It helped me to relate my theory knowledge with the clinical scenario in an actual patient and built many other attributes of a good doctor including patient interaction, counseling, and professionalism. We did group discussions, role play, demonstration, and problem-based learning to make our practical classes effective.

I used various study materials beyond textbooks including reference books, medical videos including Osmosis,^4^, Khan Academy^5^, and many more to understand the concept. I developed a habit of studying every day, practicing problem-based questions, solving objective multiple-choice questions to test my understanding after completing every chapter.

## PROBLEMS FACED BY FINAL YEAR MEDICAL STUDENTS AND COPING MECHANISMS

Most of the final year medical students experience stress, anxiety, sleep deprivation, mental as well as physical exhaustion, and pressure to not only complete the syllabus but also to revise multiple times to memorize it, especially during exams. Most of them also experience the imposter phenomenon, pressure to keep up with peer group which at times becomes overwhelming.

It is very essential to take small study breaks to recharge our mental and physical potential during the learning process. Sharing problems with friends and family helps to deal with stress, anxiety and overcome the imposter phenomenon. Learning mechanisms like self-regulated learning, active learning, problem-based learning, spaced repetition, and active recall can be implemented for effective learning.

## POST-COVID ERA

World Health Organization (WHO) declared the Corona Virus disease 2019 (COVID-19) outbreak a pandemic on 11th March 2020.^6^ The Nepal government implemented a nationwide lockdown since 24 March 2020 to curtail the spread of the coronavirus.^7^ All educational institutions were closed including medical schools to reduce the disease transmission and 'flatten the curve'. Clinical rotations were canceled and the medical students had to adapt to the new norm.^8^

## MY LEARNING EXPERIENCE

As the Nepal government extended the lockdown, studying at home without attending the lectures and clinical rotations became a new routine. This experience has both bright and dark sides. There was less study stress as I could take breaks more often since there was no such rush to complete the course quickly. This allowed me to implement various learning techniques like self-regulated learning, spaced repetition, and active recall. I could also read scientific articles and non-medical books; attend various online webinars on medical topics. Furthermore, I could spend more time with my family; get involved in other activities like exercise, yoga, and meditation, watch movies, pursue my hobbies including art and music. I also volunteered in organizing a fundraising program named “Fighting for Front liners” via our college-based social service club, to distribute personal protective equipment to the frontliners during the pandemic.

However, the feeling of uncertainty due to the extension of lockdown multiple times was quite frustrating at times. To overcome this, I used to share my problems with my parents and friends, watch various motivational videos to keep up with studying regularly.

## ADAPTING TO THE UNEXPECTED

With the lockdown being extended multiple times, there was uncertainty about the reopening of the medical colleges. Owing to the vast undergraduate medical syllabus and the pressure to complete it within the academic calendar, medical colleges in Nepal shifted to online classes by making use of software such as Zoom,

Google meet, Microsoft teams, Google classroom, Skype, etc.^9^ As this technique was naive to every one of us, neither students nor teachers were prepared for a sudden shift from traditional classroom learning to e-learning without any extensive planning and faculty training.

Even after the lockdown was lifted and the practical classes were also resumed, clinical rotations and bedside teaching in an actual patient were replaced by interactive lectures and case-based discussion to reduce the risk of spread of the virus.

As we had virtual classes during the last semester, we couldn't experience the hands-on training including patient interaction and thus were unable to polish the soft skills of medical practice which is crucial to a doctor. However, the practical exams were to be conducted traditionally. This pandemic gap from clinical practice had hampered our self-confidence and practical skills. To overcome this, we voluntarily visited the hospital wards to practice the skills of history taking and physical examination during a week of preparation break before exams. This helped us built confidence for the final examination. Role play, demonstration, and group discussions among peer groups also helped us to practice clinical skills and prepare for the exams.

## PROBLEMS FACED BY MEDICAL STUDENTS AND COPING MECHANISMS

The pandemic has been problematic to every individual; however, the situation has been critical for final year medical students as they were in the process of sitting for their final assessments.

Firstly, the extension of lockdown multiple times caused a sense of uncertainty leading to frustration and lack of motivation to study among medical students. In such times, sharing problems with family and friends can help students to overcome these problems. Most importantly, nutrition, sleep, and exercise should be focused on during such a crisis. To keep up with regular studying habits during the lockdown, making a timetable using Google calendar, Microsoft Outlook calendar, or Apple calendar and following it; providing rewards to own self after completion of certain milestones; watching motivational videos; using the Pomodoro technique; taking small study breaks are also helpful. Utilization of various e-learning tools which were provided free of cost by renowned institutes during lockdown period, reading online medical journals can also be done besides reading the medical textbooks.

Secondly, online classes were not as effective as bedside teaching. The barriers to virtual learning are due to time constraints, poor technical skills, poor infrastructure, frequent interruption in internet connection, lack of institutional strategies, inadequate teacher-student interaction, and a general negative attitude towards the huge shift in education methods.^10^ Online classes can be made effective if these barriers are looked upon.

Pedagogical modernization involving technology and simulation-based teaching (mannequin simulation, virtual patients, online lectures, video case vignettes, webcasting, virtual group discussions, and role play) needs to be brought to the forefront.^11^ Artificial intelligence can also be introduced to fill the gap between knowledge and clinical skills during the COVID-19 era and beyond as well.^12^

This pandemic has highlighted the significance of technical knowledge in modern medical education. It has taught us that we need to restructure the medical educational system to make it safe, sustainable, and equipped for all kinds of unexpected scenarios in the future. Creating a combined method by including both the traditional direct-in person learning with online virtual learning will offer the best of both methods individually and will be more durable and sustainable in the long run.^13^

To sum up, being a final year medical student in the past year, I could experience both the pre and post-COVID changes in the medical and personal aspects of a medical student and an aspiring doctor. However hard I had planned to make the best of the clinical year, the unforeseen happened. This lead to the extension of the one-year course of the final year to more than one and a half years; and a change in my entire plan in the second half of the year. This pandemic not only taught me adaptability but also resilience and making the best learning from even the worst of situations.

## WAYS FORWARD

Since the pandemic eras tend to recur over time and epidemics will continue to break out, we need to restructure the educational system to continue providing both knowledge and skills to medical students that would be safe and sustainable in the long run. A middle ground should be reached between complete virtual learning and bedside teaching rather than advocating for each option separately amid pandemics.

This public health crisis has demonstrated the significance of resilience and adaptability in the medical education system and the need to inculcate these same values in our generation of medical students. This will also help the students to complete their transition from a 'student' to a 'doctor'.

## References

[1] Shetty PA, Magazine R, Chogtu B (2020). Patient outlook on bedside teaching in a medical school.. J Taibah Univ Med Sci..

[2] Sasidharan S, Dhillon HS, Singh S (2020). Medical pedagogy in the time of COVID-19.. Kathmandu Univ Med J (KUMJ)..

[3] Nair BR, Coughlan JL, Hensley MJ (1997). Student and patient perspectives on bedside teaching.. Med Educ..

[4] Osmosis [Internet] (2012). https://www.youtube.com/channel/UCNI0qOojpkhsUtaQ4_2NUhQ.

[5] Khan Academy [Internet] (2006). https://www.youtube.com/user/khanacademy.

[6] Cucinotta D, Vanelli M (2020). WHO declares COVID-19 a pandemic.. Acta Biomed..

[7] Kathmandu Post (2020). Government extends lockdown until May 18 [Internet].

[8] Sigdel S, Ozaki A, Dhakal R, Pradhan B, Tanimoto T (2021). Medical education in Nepal: Impact and challenges of the COVID-19 pandemic.. Acad Med..

[9] Nepal S, Atreya A, Menezes RG, Joshi RR (2020). Students' Perspective on online medical education amidst the COVID-19 pandemic in Nepal.. J Nepal Health Res Counc..

[10] Sahi PK, Mishra D, Singh T (2020). Medical education amid the COVID-19 pandemic.. Indian Pediatr..

[11] Saiyad S, Virk A, Mahajan R, Singh T (2020). Online teaching in medical training: establishing good online teaching practices from cumulative experience.. Int J Appl Basic Med Res..

[12] Abhee SS, Phillips R (2020). How artificial intelligence (AI) could have helped our medical education during the COVID-19 pandemic - A student's perspective.. Med Teach..

[13] Althwanay A, Ahsan F, Oliveri F, Goud HK, Mehkari Z, Mohammed L (2020). Medical education, pre- and post-pandemic era: A review article.. Cureus..

